# The effect of ethnicity on outcomes in a practice-based trial to improve cardiovascular disease prevention

**DOI:** 10.1186/1475-9276-3-12

**Published:** 2004-12-07

**Authors:** Paul J Nietert, Steven M Ornstein, Ruth G Jenkins, Loraine F Roylance, Lori M Dickerson, Chris Feifer

**Affiliations:** 1 Department of Biostatistics, Bioinformatics, and Epidemiology, Medical University of South Carolina, 135 Cannon St., Suite 403, P.O. Box 250837, Charleston, SC 29425 USA; 2 Department of Family Medicine, Medical University of South Carolina, 295 Calhoun Street, Charleston, SC 29425 USA; 3 Department of Family Medicine, University of Southern California, 1000 S. Fremont Ave., Bldg. A7, Rm. 7419, Alhambra, CA 91803 USA

## Abstract

**Background:**

Health disparities are a growing concern. Recently, we conducted a practice-based trial to help primary care physicians improve adherence with 21 quality indicators relevant to the primary and secondary prevention of cardiovascular disease and stroke. Although the primary concern in that study was whether patients in intervention practices outperformed those in control practices, we were also interested in determining whether minority patients were more, less, or just as likely to benefit from the intervention as non-minorities.

**Methods:**

Baseline (fourth quarter 2000) and follow-up (fourth quarter 2002) data were obtained from 3 intervention practices believed to have at least 10% minority representation. Two practices had a black (non-Hispanic) population sufficient for analysis, while the other had a sufficient Hispanic population. Within each practice, changes in the 21 indicators were compared between the minority patient population and the entire patient population. The proportion of measures in which minority patients exhibited greater improvement was calculated for each practice and for all 3 practices combined, and comparisons were made using non-parametric methods.

**Results:**

For all black patients, the observed improvement in 50% of 22 eligible study indicators was better than that observed among all white patients in the same practices. The average changes in the study indicators observed among the black and white patients were not significantly different (p = 0.300) from one another. Likewise for all minority patients in all 3 practices combined, the observed improvement in 14 of 29 (43.3%) eligible study indicators was better than that observed among all white patients. The average changes in the study indicators among all minority patients were not significantly different from the changes observed among the white patients (p = 0.272).

**Conclusions:**

Among 3 intervention practices involved in a quality improvement project, there did not appear to be any significant disparity between minority and non-minority patients in the improvement in study indicators.

## Introduction

In 2002, the Institute of Medicine (IOM) issued a report suggesting that minorities are more likely than non-minorities to receive a lower quality of healthcare [1]. Because of the issues such as those raised in the IOM report, health disparities are a growing concern. This concern is reflected in many ways, including the development by National Institutes of Health of a program of action to confront these disparities and the Healthy People 2010 goal of eliminating these disparities.

Disparities are particularly evident in the area of chronic diseases. Although blacks are more likely than whites to have blood pressure monitoring, cholesterol screening, and smoking counseling, coronary heart disease is more prevalent among blacks than among whites [2]. Additionally, among all ethnic groups, blacks experience the highest mortality rates associated with heart disease, cancer, cerebrovascular disease, and HIV/AIDS. Although the overall mortality rate among blacks has been declining over the past 50 years, rates for cancer and diabetes were actually higher in 1995 than in 1950. On a similar note, Hispanics are significantly more likely as non-Hispanic whites to die from diabetes and HIV/AIDS [3].

In hopes of improving health outcomes and prevention practices for all patients, much focus has recently been given towards the improvement in quality of healthcare. For example, the 7^th ^report of the Joint National Committee on Prevention, Detection, Evaluation, and Treatment of High Blood Pressure outlines specific guidelines for preventing and managing hypertension, hyperlipidemia, and coronary heart disease [4]. The 2nd report of the National Cholesterol Education Program (NCEP) expert panel on detection, evaluation, and treatment of high blood cholesterol in adults makes specific recommendations on the prevention and care of hyperlipidemia and coronary heart disease [5]. Other quality indicators also exist for the prevention and management of other chronic diseases, including heart failure, atrial fibrillation, and diabetes, diseases which were all targeted in the current study.

Recently, there have also been a number of practice-based interventions aimed at improving the quality of healthcare for patients. For example, researchers have shown that a practice-based intervention (the Healthy Steps for Young Children Program) can enhance the quality of care for families of young children [6]. Additionally, a practice-based telephone intervention was proven to improve pneumococcal vaccine immunization for seniors [7]. We have also reported on a practice-based intervention to help primary care physicians improve adherence with 21 quality indicators relevant to the primary and secondary prevention of cardiovascular disease and stroke [8,9].

What these earlier interventions have lacked, however, are analyses examining whether the interventions have improved the quality of care for all patients, regardless of ethnicity. Because these types of interventions are heavily dependent on physician and/or clinical staff interaction with patients, because ethnic minorities may have less trust in their healthcare providers [10], and because barriers in the patient physician relationship may contribute to the ethnic disparities in the quality of the healthcare experience [11], there exists the possibility that poor cultural competency could result in a lack of effectiveness of the intervention among ethnic minorities. If such quality improvement efforts do not improve care for all ethnic groups equally, then there may be significant healthcare policy implications related to the refinement of existing interventions and to the development of future interventions.

The aim of this study was to examine whether or not a multi-method quality improvement (QI) intervention was equally successful among patients of different ethnicities. Some of the findings from this QI intervention have been previously published [8,12], and they suggest that primary care practices that use electronic medical records and receive regular performance reports can improve their adherence with clinical practice guidelines for cardiovascular disease and stroke prevention.

## Methods

The multi-method QI intervention added practice site visits (for academic detailing and QI facilitation) and network meetings (for sharing of best practices) to the approach of guideline dissemination and audit and feedback, employed in a less intensive intervention. Ten sites received the intensive multi-method QI intervention, and ten sites received the less intensive intervention. The study was conducted in a practice-based research network (PPRNet) among users of a common electronic medical record (Practice Partner Patient Records, Seattle WA), which historically provided audit and feedback to its practice members.

As a supplement to the original study, we were also interested in whether minority patients were more, less, or just as likely to benefit from the intervention as non-minorities. The study presented here focused on outcome and process measures for minorities within 3 primary care practices, all of which received the intensive intervention. These 3 practices (labeled A, B, and C) were selected because they each had a significant (i.e. > 10%) proportion of minority patients and had recorded patient ethnicity in their electronic medical record. Practice A is an urban internal medicine practice in the Midwestern U.S. with 5 healthcare providers. Practice B is a rural family medicine practice in the Northeastern U.S. with 8 healthcare providers. Practice C is an urban family medicine practice in the Southeastern U.S.

A total of 21 study indicators (see Table 1) were obtained from each practice at baseline (fourth quarter 2000) and at the end of the study (fourth quarter 2002). These indicators were derived from published sources [4,5,13-16] and were deemed to be the most appropriate indicators for measuring quality of prevention and management of cardiovascular disease and stroke. Fourteen of the study indicators are process measures, reflecting whether recommended tests were done, appropriate diagnoses made or medication prescribed. Seven indicators are outcome measures, reflecting whether patients achieved recommended treatment goals. Some of the measures represent primary prevention, e.g., screening for hypertension or hyperlipidemia. Others represent secondary prevention, e.g., reaching treatment goals for glycosylated hemoglobin, low-density lipoprotein (LDL) cholesterol, and blood pressure in patients with diabetes. The institutional review board at the Medical University of South Carolina approved the study.

**Table 1:** Study indicators

CONDITION	MEASURES
Hypertension^1^	Process measures:
	• BP measurement in prior 12 months
	• Diagnosis of hypertension for 3 measurements >= 140/90 in prior 12 months
	• BP measurement in prior 3 months for patients with diagnosis of hypertension
	Outcome measures:
	• Most recent BP measurement < 140/90 for all patients
	• Most recent BP measurements < 140/90 for patients with diagnosis of hypertension
Hyperlipidemia (General Population screening)^2^	Process measures:
	• Measure of total cholesterol in prior 36 months
	• Measure of HDL-C in prior 36 months
Coronary Heart Disease ^1,2,3^	Process measures:
	• Measurement of LDL-cholesterol in prior 12 months
	• Recorded diagnosis of hyperlipidemia for LDL-cholesterol > 130 mg/dl
	• Medication for hyperlipidemia for LDL-cholesterol > 130 mg/dl
	• Prescription of beta-blocker in patients with history of myocardial infarction
	Outcome measures:
	• Most recent LDL-cholesterol < 100 mg/dl
	• Most recent BP measurement < 140/90
Heart Failure^4^	Process measure:
	• Prescription of angiotensin converting enzyme inhibitor or angiotensin receptor blocker
Atrial Fibrillation^5^	Process measure:
	• Prescription of oral anticoagulant
Diabetes Mellitus^6^	Process measures:
	• Measurement of glycosylated hemoglobin in prior 12 months
	• Measurement of LDL-cholesterol in prior 24 months
	• BP measurement in prior 3 months
	Outcome measures:
	• Most recent glycosylated hemoglobin < 7 %
	• Most recent LDL-cholesterol < 100 mg/dl
	• Most recent BP measurement < 130/85

^1 ^Adapted from The Sixth Report of the Joint National Committee on Detection, Evaluation and Treatment of High Blood Pressure (JNC VI). National Institute of Health, National Blood Pressure Education Program, NIH publication 98-4080, November 1997.

^2 ^Adapted from Summary of the Second Report of the National Cholesterol Education Program (NCEP) Expert Panel on Detection, Evaluation, and Treatment of High Blood Cholesterol in Adults (Adult Treatment Panel II). JAMA 1993; 269(23): 301523.

^3 ^Adapted from American Heart Association Scientific Statement: Smith SC, Blair SN, Criqui MH, etal: Preventing Heart Attack and Death in Patients with Coronary Disease. *Circulation*. 1995;92:2–4. Although the AHA recommendation is to measure total cholesterol at least every 60 months, we were forced to restrict our measure to 36 months due to restrictions of the historical data.

^4 ^Adapted from American Heart Association Scientific Statement: Williams JF, Bristow MR, Fowler MB, etal: Guidelines for the Evaluation and Management of Heart Failure. *Circulation*. 1995;92:2764–2784

^5 ^Adapted from American Heart Association Scientific Statement: Prystowsky EN, Benson DW, Fuster, V, etal: Management of Patients with Atrial Fibrillation. *Circulation *1996;93:1262–1277.

^6 ^Adapted from American Diabetes Association, Diabetes Quality Improvement Project, Initial Measure Set (Final Version) August 14, 1998

To determine practice performance on the study indicators, participating practices ran a computer program to extract patient activity during the previous quarter from their electronic medical record. To protect patient confidentiality, the extract program assigned an anonymous numerical identifier unique to each patient. The extract program obtained demographic information such as age, ethnicity, and gender, and diagnoses, medications, laboratory data, and vital signs. Text of consultation reports, progress notes, and discharge summaries were not extracted. The data were copied to diskettes and mailed to PPRNet or sent electronically via a secure server. In the PPRNet offices, data were bridged to standard data dictionaries and converted to SAS^® ^(Statistical Analysis System, Cary NC) data sets on standard microcomputers for analyses.

In each patient's electronic medical record, ethnicity was recorded as white, black/African American, American Indian/Alaskan native, Asian, native Hawaiian/other Pacific islander, and "some other ethnicity", while ethnicity was recorded as Hispanic/Latino and non-Hispanic/Latino, all in concordance with the 2000 U.S. Census ethnicity categories. Currently, these physician practices allow the patient to designate their ethnicity categorization. However, because this process for collecting ethnicity data began in the middle of our study, some ethnicity categorizations were made by the office staff within each of the practices. Ethnicity data was only available on approximately 42% of patients, due to the fact that the electronic medical record software program did not require physicians to enter patients' ethnicity data until its most recent version was released, which occurred during the study time frame. Improvements in process and outcome measures were compared between minority and non-minority patients. Minority was defined as any ethnic designation other than white non-Hispanic.

Changes in the process and outcome measures were of primary interest in this study. Within each practice, these changes were compared between the minority patient population and the white patient population. Measures were deemed eligible for comparison if at least 10 minority patients were included in the rate calculations. For example, if practice A only had 8 minority patients with a diagnosis of having had myocardial infarction (MI), then the measure of the percentage of MI patients who had been prescribed a beta blocker could not be compared between the minority and white patient population. The proportion of eligible measures in which minority patients exhibited greater improvement was calculated for each practice and for all 3 practices combined. A Wilcoxon signed rank test (the non-parametric equivalent of the paired t-test) was used to test the hypotheses that minority patients exhibited changes similar to those of the non-minority patients. This study had approximately 80% power (2-sided hypothesis testing, α = 0.05) to detect a 6.6 percentage point difference between average improvement in the study indicators among all minority and non-minority patients.

## Results

Baseline characteristics of the patients from the 3 practices are listed in table 2. In practice A, black (non-Hispanic) patients were the only sizable minority. Although practice B did contain 10 black non-Hispanic patients, this sample was not large enough for substantive comparisons. There were enough Hispanic patients in Practice B to compare with the entire groups of patients within that practice. In practice C, there were 117 black patients used for comparison. There were several significant differences of note between the minority patients to the overall population of patients within that same practice. Compared to the white patient population in practice A, the minority patients were significantly younger and significantly less likely to be diagnosed with hyperlipidemia. Compared to the white patient population in practice B, the minority patients were significantly more likely to be male and to have diabetes. In practice C, the minority patients were significantly younger, more likely to be female, and less likely to have a diagnosis of hyperlipidemia.

**Table 2:** Baseline characteristics of the patients within the 3 practices of interest

Characteristic	Practice A		Practice B		Practice C	
	Black patients (n = 179)	White patients (n = 1,079)	Hispanic patients (n = 254)	White patients (n = 2,526)	Black patients (n = 117)	White patients (n = 491)
Demographics						
Age (mean ± s.d.)	54.2 ± 15.2****	60.7 ± 17.8	34.5 ± 17.9	33.7 ± 18.9	42.0 ± 16.7***	51.2 ± 16.9
Gender (% female)	68.7	69.2	46.1***	57.9	67.5*	56.4
Medical conditions						
Hypertension (%)	50.8	51.1	7.1	7.8	21.4	21.0
Hyperlipidemia (%)	33.5***	47.5	5.5	4.1	0.9*	5.3
Diabetes (%)	12.9	9.7	4.3*	2.0	12.0	7.5
Coronary disease (%)	7.8	11.7	1.2	1.0	0.0	0.0
Heart failure (%)	2.2	4.9	0.0	0.2	1.7	0.6
Atrial fibrillation (%)	1.1	3.9	0.0	0.1	0.0	0.2

* p < 0.05 when compared to white patients within the particular practice

** p < 0.01 when compared to white patients within the particular practice

*** p < 0.001 when compared to white patients within the particular practice

**** p < 0.0001 when compared to white patients within the particular practice

Additional file 1 lists the baseline and end-of-study measurements for each of the 21 study indicators, for minority patients and white patients within each of the 3 practices. In practice A, the improvement in 7 of 16 eligible study indicators was better among black patients than among white patients in that practice. (For 9 of these 16 indicators, the improvement was worse among the black patients.) In practice B, the improvement in 3 of 7 eligible study indicators was better among Hispanic patients than among white patients in that practice, and worse for 4 of the 7 indicators. In practice C, the improvement in 4 of 6 eligible study indicators was better among black patients than among white patients in that practice. Thus for all black patients in practices A and C, the observed improvement in 11 of 22 (50.0%) eligible study indicators was better than that observed among white patients. On average, indicators improved 4.4 and 9.3 percentage points among black and white patients, respectively. These changes were not significantly different (p = 0.300) from one another. Likewise for all minority patients in all 3 practices combined, the observed improvement in 14 of 29 (48.3%) eligible study indicators was better than that observed among non-minority (white) patients. On average, indicators improved 4.6 and 8.3 percentage points among minority and non-minority patients, respectively, and these changes were not significantly different (p = 0.272) from one another.

## Discussion

In these 3 physician practices, all of which were in the intervention arm of a randomized trial aimed at improving primary and secondary prevention of cardiovascular disease and stroke, we found that results for minorities were relatively similar to the results experienced by the overall practice populations. Change from baseline was greater among minority patients than among white patients for 48.3% of the 29 eligible study indicators, and the average changes in the study indicators among all minority patients were not significantly different from the changes observed among the white patients.

There are some limitations of this study which should be noted. As noted earlier, the ethnicity status was only available on 42% of patients within the practices of interest; thus the results may not truly represent what occurred in these practices overall during the study. Given the relatively small number of eligible indicators for comparisons across ethnicities, this statistical power to detect subtle differences was not optimal. Nevertheless, the overall findings suggest that any true differences in this intervention's effectiveness across ethnicities are small.

These findings are encouraging, and they suggest that the quality improvement strategies that have been developed to date for physician practices that use electronic medical records have a similar impact on minorities and non-minorities. Future studies should continue to address whether the effectiveness of interventions such as ours is cross-cultural, and whether interventions tailored to be more culturally appropriate can improve the overall effectiveness of interventions.

## List of abbreviations used

IOM: Institute of Medicine

HIV: Human Immunodeficiency Virus

AIDS: Acquired Immunodeficiency Syndrome

QI: Quality Improvement

LDL: Low-Density Lipoprotein

MI: Myocardial Infarction

## Competing Interests

The author(s) declare that they have no competing interests.

## Authors' Contributions

PJN helped design the study, perform the analyses, and write the manuscript. SMO helped design the study, perform the site visits, and edit the manuscript. RGJ helped design the study, perform the analyses, and write the manuscript. LFR helped design the study, assisted with data acquisition, and edited the manuscript. LMD helped design the study, perform the site visits, and edit the manuscript. CF helped perform site visits and edit the manuscript. All authors read and approved the final manuscript.

## Supplementary Material

Additional File 1 Study indicators as measured at baseline (B) and the end (E) of the study for all patients and minority patients within each of the 3 practices. Click here for file
