# Supplementary material for: The effect of ethnicity on outcomes in a practice-based trial to improve cardiovascular disease prevention
Source: Int J Equity Health. 2004 Dec 7;3:12. doi: 10.1186/1475-9276-3-12 (PMC544361; doi:10.1186/1475-9276-3-12)
Supplement: Additional File 1 — Study indicators as measured at baseline (B) and the end (E) of the study for all patients and minority patients within each of the 3 practices. [file 1475-9276-3-12-S1.pdf]

Table 3. Study indicators as measured at baseline (B) and the end (E) of the study for all patients and minority patients within each of the 3 practices.

Study Indicator	Practice A				Practice B				Practice C			
	Black Patients		White Patients		Hispanic Patients		White Patients		Black Patients		White Patients	
	B	E	B	E	B	E	B	E	B	E	B	E
Hypertension												
BP in 12 months	90.5%	92.5%*	89.6%	89.8%	85.0%	87.8%*	88.0%	87.6%	97.3%	99.1%*	98.1%	99.2%
Dx HTN 3 BP >=140/90	88.9%	97.1%*	88.8%	94.8%	-	-	56.1%	68.3%	-	91.7%	88.9%	81.5%
BP in 3 months HTN patients	63.7%	67.9%*	61.5%	65.6%	38.9%	33.3%*	67.5%	48.0%	56.0%	60.0%*	56.3%	53.9%
Last BP <140/90 all patients	54.9%	70.4%	56.9%	76.0%	78.3%	87.2%	73.0%	83.2%	86.4%	96.7%*	87.9%	90.7%
Last BP <140/90 HTN patients	38.6%	64.0%	44.5%	72.0%	33.3%	50.0%	32.3%	59.5%	60.0%	63.2%	65.7%	72.4%
Hyperlipidemia												
Cholesterol in 60 months	74.3%	86.0%*	80.0%	87.7%	-	75.1%	-	66.8%	-	38.9%	-	40.0%
HDL-C in 60 months	72.1%	85.0%*	77.0%	85.6%	-	74.7%	-	67.2%	-	38.5%	-	40.0%
Coronary Heart Disease												
LDL-C in 12 months	85.7%	92.9%*	83.3%	84.2%	-	-	41.7%	61.9%	-	-	-	42.9%
Dx hyperlipidemia LDL-C > 130	-	-	96.7%	94.7%	-	-	-	-	-	-	-	-
Rx for LDL-C >130	-	-	73.3%	52.6%	-	-	-	-	-	-	-	-
Beta-blocker MI patients	-	-	-	-	-	-	-	-	-	-	-	-
Last LDL-C <100	50.0%	46.2%	34.3%	53.6%	-	-	40.0%	46.2%	-	-	-	-
Last BP <140/90	84.6%	71.4%	49.2%	80.9%	-	-	52.4%	66.7%	-	-	-	-
Heart Failure												
Rx for ACE or ARB	-	-	66.0%	71.7%	-	-	-	-	-	-	-	69.2%
Atrial Fibrillation												
Rx of oral anti-coagulant	-	-	47.6%	58.3%	-	-	-	-	-	-	-	-
Diabetes Mellitus												
HgbA1c in 12 months	82.6%	81.3%	60.7%	90.4%	-	92.9%	-	67.0%	-	40.9%	-	44.4%
LDL-C in 24 months	82.6%	90.6%*	86.9%	94.9%	45.5%	78.6%*	56.9%	80.2%	-	59.1%	-	68.1%
BP in 3 months	73.9%	75.0%	70.5%	72.1%	45.5%	21.4%	68.6%	52.8%	64.3%	59.1%*	48.7%	43.1%
Last HgbA1c < 7	63.2%	46.2%	56.8%	48.7%	-	38.5%	-	55.7%	-	-	-	-
Last LDL-C < 100	35.3%	46.4%	40.0%	56.3%	-	-	25.0%	40.0%	-	-	-	40.0%
Last BP <130/85	43.5%	65.6%	44.8%	78.8%	63.6%	66.7%	54.2%	64.4%	78.6%	68.2%	77.8%	73.2%

Abbreviations

BP: blood pressure; Dx: diagnosis; HTN: hypertension; HDL-C: high density lipoprotein cholesterol; LDL-C: low density lipoprotein cholesterol; MI: past history of myocardial infarction; Rx: prescription; ACE: angiotensin converting enzyme inhibitor; ARB: angiotensin receptor blocker; HgbA1c: hemoglobin A1c

Note: When the number of subjects for the denominator of a given measure was < 10, or if sufficient lab data were not recorded to calculate the measure of interest, the data are not considered reliable and thus indicated by a dash in the above table.

* Improvement (end-of-study – baseline) in minority group was greater than that observed for all patients.
